# Characteristics of Young Adult Defendants with Neurodevelopmental Disorders

**DOI:** 10.1192/j.eurpsy.2026.10949

**Published:** 2026-07-31

**Authors:** J. McCarthy, E. Chaplin

**Affiliations:** 1 King’s College London; 2 London South Bank University, London, United Kingdom

## Abstract

**Introduction:**

The provision of screening, legal accommodations, and diversion programmes vary across the criminal justice systems of Europe in how they identify and support young people with neurodevelopmental disorders (NDD). In some jurisdictions, young offenders with NDD may receive alternative sentencing, such as community-based interventions or specialised care programmes, while others may face standard criminal procedures. However there is little published data specifically on young adult defendants with NDD to understand their mental health needs and other vulnerabilities.

**Objectives:**

This study examines the mental health needs of young adult defendants aged between 18-24 with neurodevelopmental disorders (NDD) and with no NDD presenting to the lower courts in London, England.

**Methods:**

The study analysed service level data taken from the National Health Service Minimum Data Set which records service activity and reflects current clinical and custody records routinely collected. Data from 1118 defendants, aged 18-24 with and without NDD accessing Court Liaison and Diversion Services across five Magistrates Courts in two London regions were examined for characteristics relating to mental disorder.

**Results:**

Of the 1118 young adult defendants in this study 87.6% (979) had no NDD and 12.4%, (139) had NDD. The no NDD group had increased rates for all mental illness including personality disorder, with schizophrenia or other delusional disorders being significantly higher than rates in the NDD group. Substance misuse rates were seen at similar rates cross both groups with a significant higher rate of alcohol misuse within the NDD groups. Although psychiatric comorbidity rates were lower in the NDD group, current self-harm behaviour and/or suicide ideation was significantly higher in those defendants with NDD.

**Conclusions:**

Young adult defendants including those with NDD face complex and significant challenges that intersect with mental health, substance/alcohol misuse and access to support services. Despite existing vulnerabilities, the needs of those defendants with NDD are frequently under recognised, particularly their elevated risk for self-harm /suicide ideation and alcohol misuse meaning tailored interventions may be overlooked

**Disclosure of Interest:**

J. McCarthy Grant / Research support from: £674,000, E. Chaplin Grant / Research support from: £674,000

